# Prognostic Value of Geriatric Nutritional Risk Index in Esophageal Carcinoma: A Systematic Review and Meta-Analysis

**DOI:** 10.3389/fnut.2022.831283

**Published:** 2022-03-25

**Authors:** Jianfeng Zhou, Pinhao Fang, Xiaokun Li, Siyuan Luan, Xin Xiao, Yinmin Gu, Qixin Shang, Hanlu Zhang, Yushang Yang, Xiaoxi Zeng, Yong Yuan

**Affiliations:** ^1^Department of Thoracic Surgery, Westchina Hospital, Sichuan University, Chengdu, China; ^2^West China Biomedical Big Data Center, West China Hospital, Sichuan University, Chengdu, China

**Keywords:** geriatric nutritional risk index (GNRI), esophageal carcinoma (EC), prognostic, weight, meta-analysis

## Abstract

Esophageal cancer (EC) is one of the most common cancers worldwide. Malnutrition often leads to poor prognosis of patients with EC. Geriatric nutritional risk index (GNRI) was reported as an objective nutrition-related risk index. We intend to comprehensively review evidence of GNRI in predicting EC prognosis. To explore the influence of GNRI on the long-term survival outcome of patients with EC, a meta-analysis was needed. We searched the Web of Science, Medline, Embase, and the Cochrane Library databases. The association between prognosis of patients with EC and GNRI was evaluated by pooling hazard ratios (HRs) and their corresponding 95% confidence intervals (CIs). The fixed model or random model method was chosen according to the heterogeneity among the studies. Totally, 11 studies with 1785 patients who met the inclusion criteria were eventually included in our meta-analysis. Comparing the lower level GNRI group and the higher level GNRI group, the pooled results showed that lower GNRI had a negative impact on overall survival (OS) (HR: 1.75, 95% CI: 1.45–2.10, *P* < 0.01) and cancer-specific survival (CSS) (HR: 1.77, 95% CI: 1.19–2.62, *P* < 0.01), indicating that lower GNRI significantly predicted poor OS. In conclusion, lower GNRI could predict the poor prognosis of patients with EC. Meanwhile, more well-designed randomized controlled trials (RCTs) are needed to confirm the findings.

## Introduction

Esophageal cancer (EC) is the tenth most common malignant tumor and also one of the most common causes of cancer death worldwide (1). It consists of two main types of esophageal squamous cell carcinoma (ESCC) and esophageal adenocarcinoma (EAC). Despite advancement in therapies of EC, the 5-year post-esophagectomy survival rate is still low with only approximately 30% (2, 3). Since the symptoms of EC in the early stage are easy to be neglected, patients often lose the optimum opportunity to get surgical therapy, so the survival outcome of patients with EC remains unfavorable (4). In recent years, impaired baseline nutrition has been considered a prognostic factor of cancer, especially gastrointestinal tumors. Malnutrition is common, particularly, in patients with upper digestive tract malignancies due to nutrition loss, increased metabolic demands, and an insufficient oral intake (5). Remarkably, it is reported that 60–80% of patients with EC suffered from malnutrition. Malnutrition is generally evaluated as low body mass index (BMI) and low level of serum albumin. Meanwhile, malnutrition is reported to be associated with poor short- and long-term clinical outcomes in patients with EC (6). Quantities of relevant studies have been conducted with mixed results. Therefore, the association between the overall survival (OS) of patients with EC and malnutrition remains still controversial.

Geriatric nutritional risk index (GNRI) was first proposed by Bouillanne et al. (7) in 2005, taking both serum albumin and the ratio of present body weight to ideal body weight into consideration. GNRI is regarded as a better indicator of nutrition-related outcomes better than serum albumin level and BMI alone in elderly patients. GNRI is calculated by the formula as follows: GNRI = (1.489 × albumin, g/L) + (41.7 × present/ideal body weight, kg) (7). It has been originally recommended for the assessment of patients, such as elderly patients with high risk for cardiovascular disease (8), hemodialysis (9), and chronic obstructive pulmonary disease (10). To date, several cohort studies but not meta-analysis studies have explored the relationship between GNRI and the OS of patients with EC. Therefore, this meta-analysis is needed to investigate the prognostic value of GNRI in patients with EC and to evaluate whether the GNRI could be used as a prognosis predictor in patients with EC.

## Materials and Methods

### Search Strategies

Systematic literature retrieval of the Embase, Medline, Web of Science, and the Cochrane Library was performed till July 1, 2021, using the following search strategies and terms: (((((((esophagus [Title/Abstract]) OR esophageal [Title/Abstract]) OR oesophagus [Title/Abstract]) OR oesophageal [Title/Abstract])) AND (((tumor [Title/Abstract]) OR cancer [Title/Abstract]) OR carcinoma [Title/Abstract])) AND (((prognostic [Title/Abstract]) OR prognosis [Title/Abstract]) OR survival [Title/Abstract])) AND (GNRI [All Fields]).

### Study Selections

The included standards were as follows: (1) study patients were pathologically confirmed EC without evidence of metastasis or recurrence; (2) observational studies or randomized controlled trials (RCTs) were eligible, which explored the effect of GNRI on the survival outcomes of patients with EC; (3) studies clearly illustrate the correlation between GNRI and survival outcomes of patients with EC; (4) the patients in studies had received treatment options such as surgery, radiotherapy, or chemotherapy; (5) the patients were grouped according to the level of GNRI; (6) papers published in English only; and (7) more than 5 points of Newcastle-Ottawa Scale (NOS) score were considered eligible for inclusion. The following studies were excluded: (1) patients with non-esophageal carcinoma; (2) article type such as case report, review, abstract, animal experiment, and conference report; (3) without sufficient data for meta-analysis; and (4) duplicated studies.

### Data Extraction and Quality Assessment

Relevant data were extracted from included studies and compared results by two authors (JZ and PF) independently. Adjudication was performed by the third author (XL) to resolve discrepancies and avoid bias. A standardized data extraction procedure was used to retrieve the data from studies. The basic characteristics of studies, including author, publication year, number of the patients, age, study design, cutoff value, treatment, and survival outcomes, were extracted. The NOS (11) was utilized to evaluate the quality and risk-of-bias of observational studies, which consisted of the following three factors: selection of patients, comparability between the groups, and assessment of interesting outcomes. Studies were assigned using a score of 0–9 (allocated as stars), and we defined 0–3, 4–6, and 7–9 as low, medium, and high quality studies, respectively.

### Statistical Analysis

Data analyses were based on STATA 12.0 package (StataCorp, College Station, TX, USA) in accordance with PRISMA guidelines (12). The survival outcome rate data were collected from the papers directly or Kaplan-Meier curves. Hazard ratio (HR) with a 95% confidence interval (CI) was adopted for the comparison. The heterogeneity of each study was evaluated by using a chi-square-based *Q*-test and the *I*^2^ test. If low heterogeneity between studies (*P*_Q_ > 0.05, *I*^2^ < 50%) was observed, a fixed-effect model would be applied for analysis. Otherwise, random-effects models were used. Sensitivity analysis by sequentially removing one study at a time was performed. The potential publication bias was estimated by a funnel plot, and Begg's test was performed to assess the asymmetry. All *P*-values were two-sided. A *P*-value < 0.05 was considered statistically significant (13).

## Results

### Characteristics of Studies

**Figure 1:**
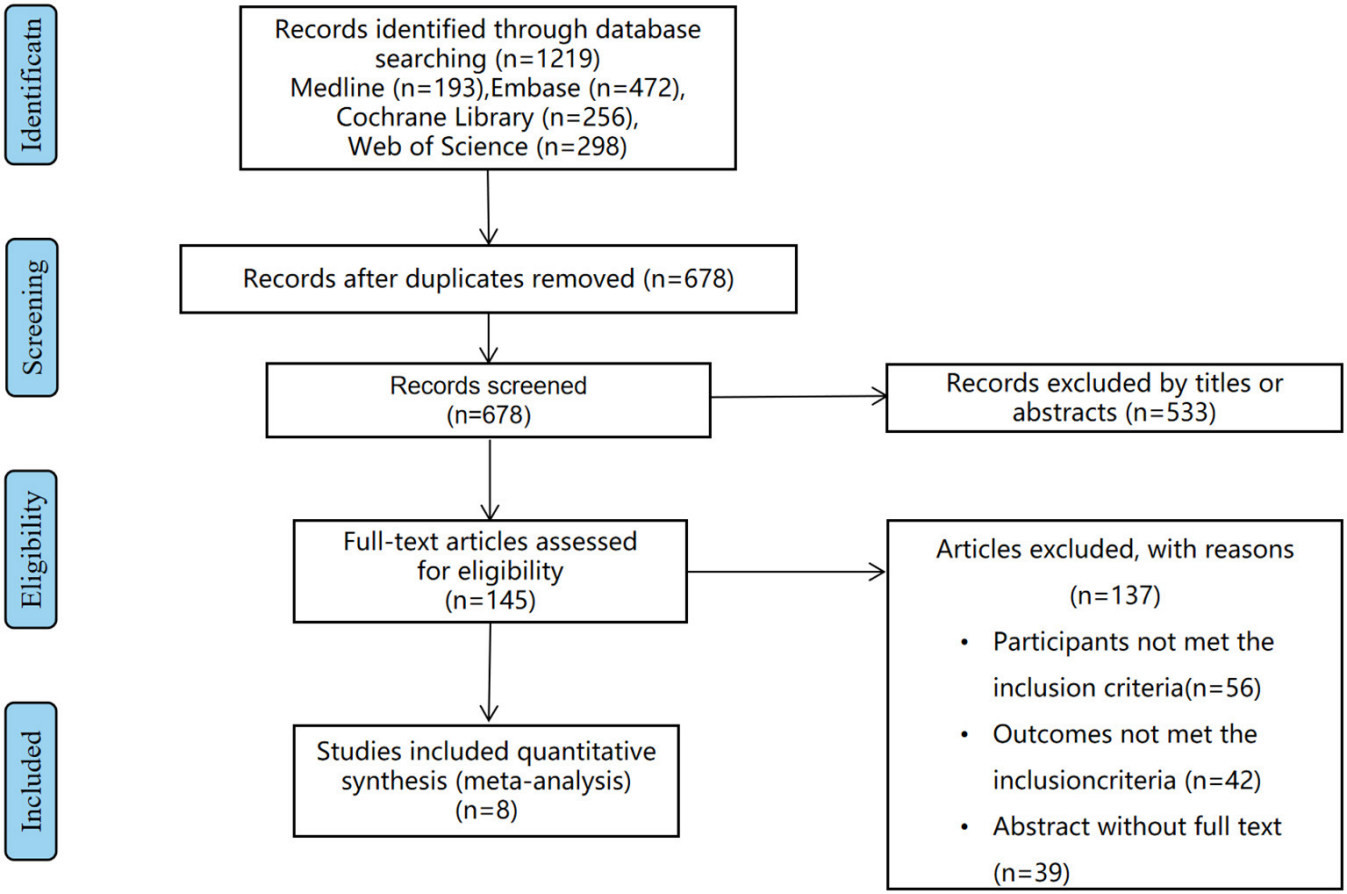
Methodological flowchart of the review.

Based on the criteria mentioned earlier, the search results are shown in Figure 1. After the initial search, 1,219 articles were found through 4 databases after the removal of 541 duplicates. Then, there were 533 studies removed after reviewing titles and abstracts. Later, 137 articles were found not meeting the inclusion criteria by further full-text screening. Eventually, 8 articles (14–21) involving 11 studies, containing 1785 patients in total, were included in the meta-analysis. All the included studies had reported the cutoff point of the GNRI, with different fixed values as follows: 92, 96.6, 97.1, and 98. As for survival outcomes, HRs on OS, cancer-specific survival (CSS) could be extracted from 9 and 2 of these studies, respectively. Notably, 448 patients underwent surgery, 862 patients underwent surgery combing oncological treatment, and 475 patients underwent non-surgery treatment. Both patients included were from Asia, China, and Japan. More detailed information and basic characteristics of the included studies in this meta-analysis are summarized in Table 1. Based on the NOS, the included studies' scores ranging from 6 to 7, showing the qualities of these studies, were high, which are eligible for the subsequent analysis. In Table 2, we specified population, expose, comparison, and outcome (PECO) elements of each study and whether it is an observational study or a secondary observational study in an interventional study.

**Table 1:** Main characteristics of all the studies included in the meta-analysis.

**Study**	**Year**	**Population**	**No. (M/F)**	**Follow-up (months)**	**Treatment**	**Age (years)**	**Cut-off**	**Outcome**	**Stage**	**Type**	**HR**	**NOS score**
Bo et al. (14)	2016	Chinese	207(130/77)	120	Radiotherapy	NR	92	OS	I-IV	ESCC	M	7
Bo et al. (14)	2016	Chinese	216(133/83)	120	Radiotherapy	NR	98	OS	I-IV	ESCC	M	7
Kubo et al. (17)	2018	Japanese	240(193/47)	60	Surgery + neoadjuvant therapy	63.4 ± 7.8	92	OS	I-IV	ESCC	M	7
Kubo et al. (17)	2018	Japanese	240(193/47)	60	Surgery + neoadjuvant therapy	63.4 ± 7.8	92	CSS	I-IV	ESCC	M	7
Migita et al. (18)	2018	Japanese	137(116/21)	60	Surgery + chemoradiotherapy	NR	98	OS	I-III	ESCC	M	6
Yamana et al. (21)	2018	Japanese	54(NR)	50	Surgery + neoadjuvant therapy	NR	92	OS	I-IV	ESCC	M	6
Yamana et al. (21)	2018	Japanese	162(NR)	50	Surgery	NR	92	OS	I-IV	ESCC	M	6
Wang et al. (19)	2019	Chinese	52(34/18)	60	Radiotherapy or definitive concurrent chemoradiotherapy	74 (70-83)	92	OS	I-IV	ESCC	M	6
Hirahara et al. (15)	2020	Japanese	191(169/22)	72	Surgery + adjuvant chemotherapy	NR	97.1	CSS	I-III	ESCC	M	7
Kouzu et al. (16)	2020	Japanese	128(113/15)	60	Surgery	73.2 ± 5.5	92	OS	I-IV	EC	U	6
Tan et al. (19)	2021	Chinese	158(126/32)	80	Surgery	70.7 ± 4.49	96.6	OS	I-IV	EC	M	6

*OS, overall survival; CSS, cancer-specific survival; HR, hazard ratio, “M” means the HR come from multivariate analysis, “U” means the HR comes from univariate analysis; NR, not reported; ESCC, esophageal squamous cell cancer; EC, esophageal carcinoma; NOS, Newcastle-Ottawa Scale*.

**Table 2:** Abstract table summarizing PECO in the studies of GNRI.

**Study**	**Population**							**Expose**	**Comparison**	**Outcome**	**Research type**	**NOS scores**
	**EC stage**				**Phase of GNRI assessment**							
	**I**	**II**	**III**	**IV**	**Pre-treatment**		**Post-treatment**					
Bo et al. (14)	22	138	54	25		NR				OS	Observational study	7
Kubo et al. (17)	70	51	105	14		NR				OS	Observational study	7
Kubo et al. (17)	70	51	105	14		NR				CSS	Observational study	7
Migita et al. (18)	NR	NR	NR	NR	√					OS	Observational study	6
Yamana et al. (21)	NR	NR	NR	NR	√			Low GNRI	High GNRI	OS	Observational study	6
Wang et al. (20)	20		32			NR				OS	Observational study	6
Hirahara et al. (15)	73	41	77	NR			√			CSS	Observational study	7
Kouzu et al. (16)	70		58		√					OS	Observational study	6
Tan et al. (19)	NR	NR	NR	NR	√					OS	Observational study	6

*PECO, population, expose, comparison, and outcome; OS, overall survival; CSS, cancer-specific survival; EC, esophageal carcinoma; GNRI, geriatric nutritional risk index; NR, not reported*.

### Meta-Analysis

**Figure 2:**
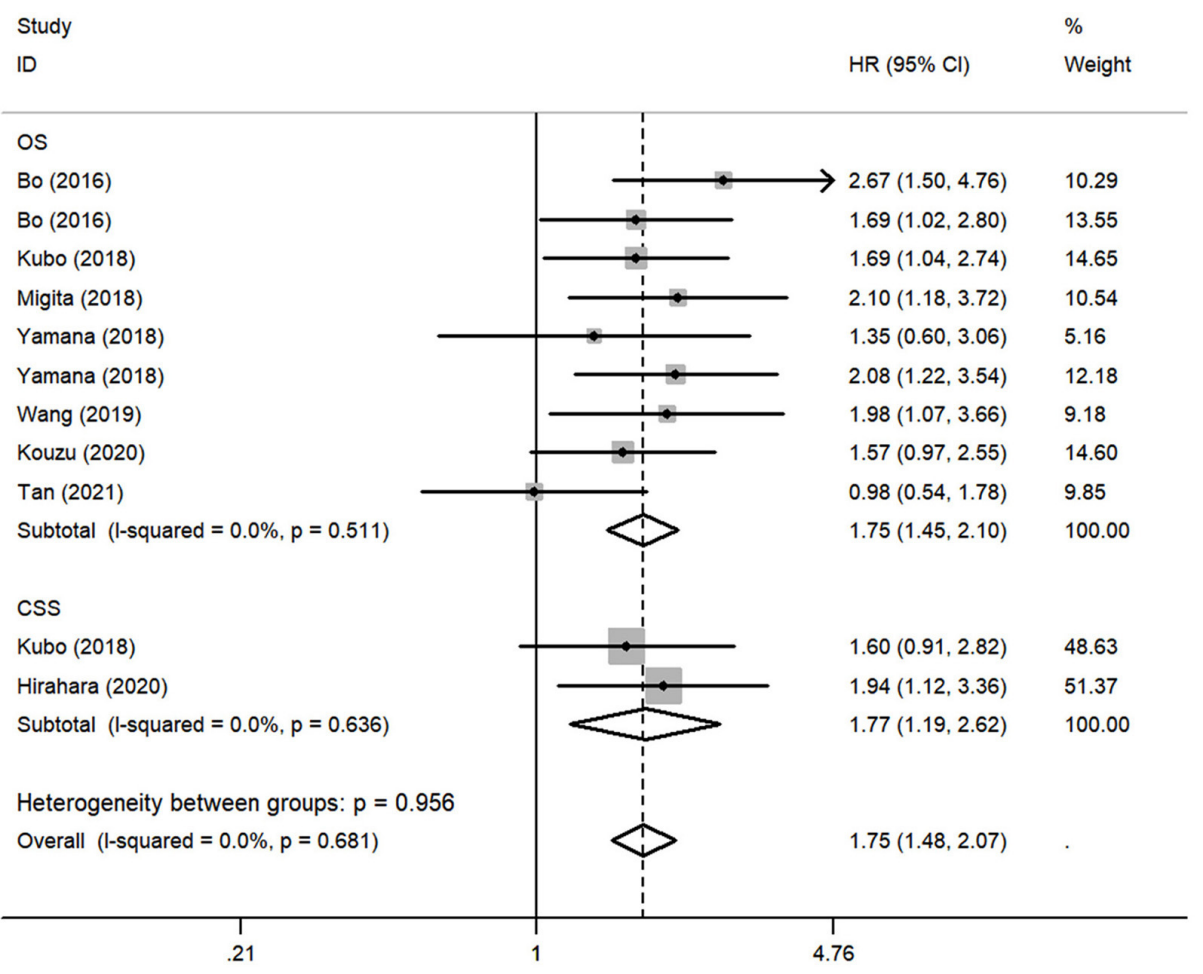
Forest plot of pooled hazard ratio (HR) of geriatric nutritional risk index (GNRI) in predicting survival outcomes in esophageal cancer (EC).

To assess the impact of lower GNRI on OS, a fixed-effects model was conducted to analyze since the heterogeneity was non-significant (*I*^2^ = 0.0%, *P* = 0.511). Totally, 9 studies contained a number of 1354 patients applying OS as the survival outcome. The pooled HR was 1.75 (95% CI: 1.45–2.10, *P* < 0.01) (Figure 2), indicating that patients with low GNRI had poorer OS than those with high GNRI. To investigate the correlation between GNRI and CSS, 2 studies with a total of 431 patients were included. Heterogeneity was acceptable in the analysis (*I*^2^ = 0%, *P* = 0.636), a fixed-effects model was used, and the pooled HR was 1.77 (95% CI: 1.19–2.62, *P* < 0.01) (Figure 2), suggesting that low GNRI was significantly associated with worse CSS.

**Figure 3:**
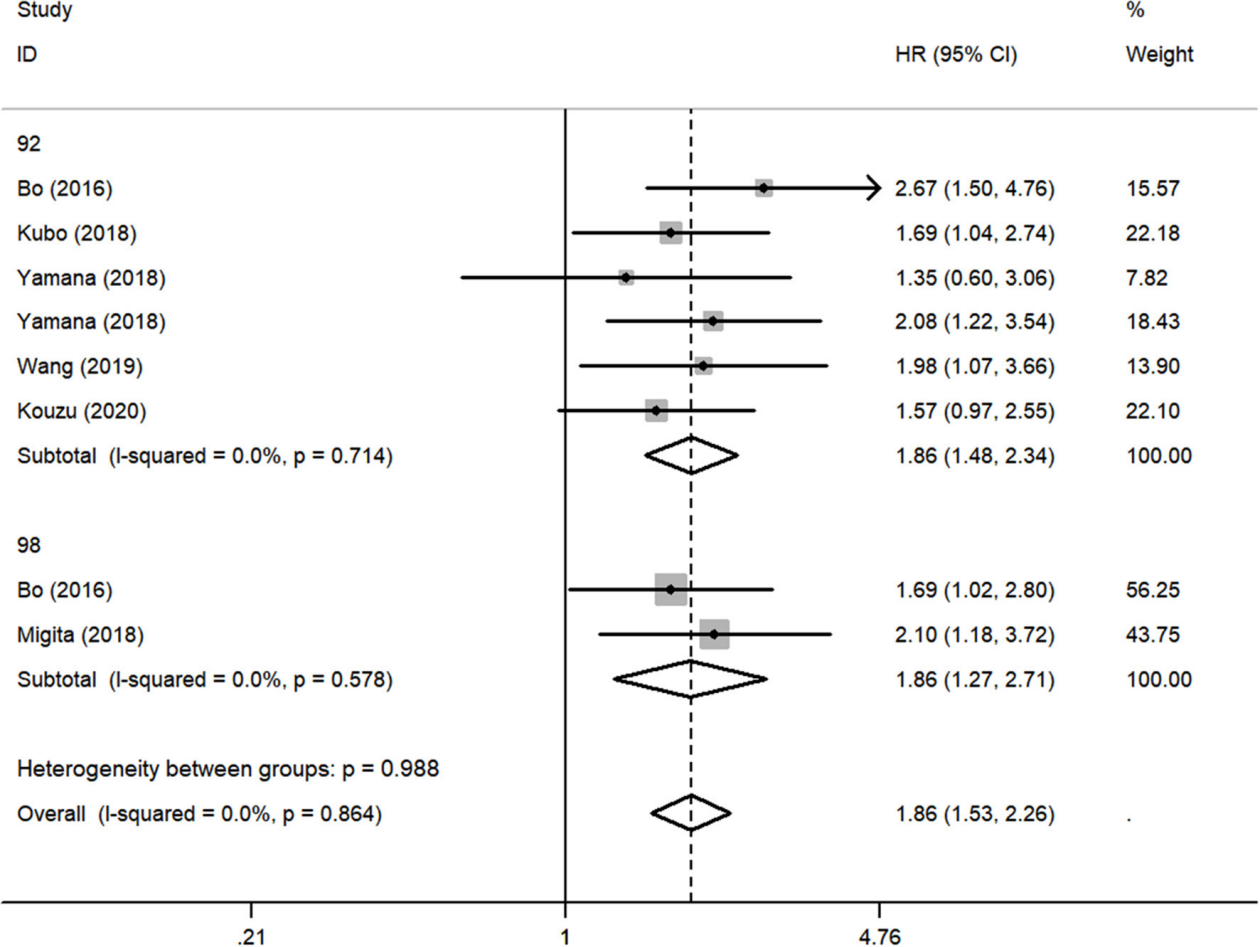
Forest plot showing subgroup analysis of the selected studies about the prognostic significance of GNRI in patients with different cutoff values.

**Figure 4:**
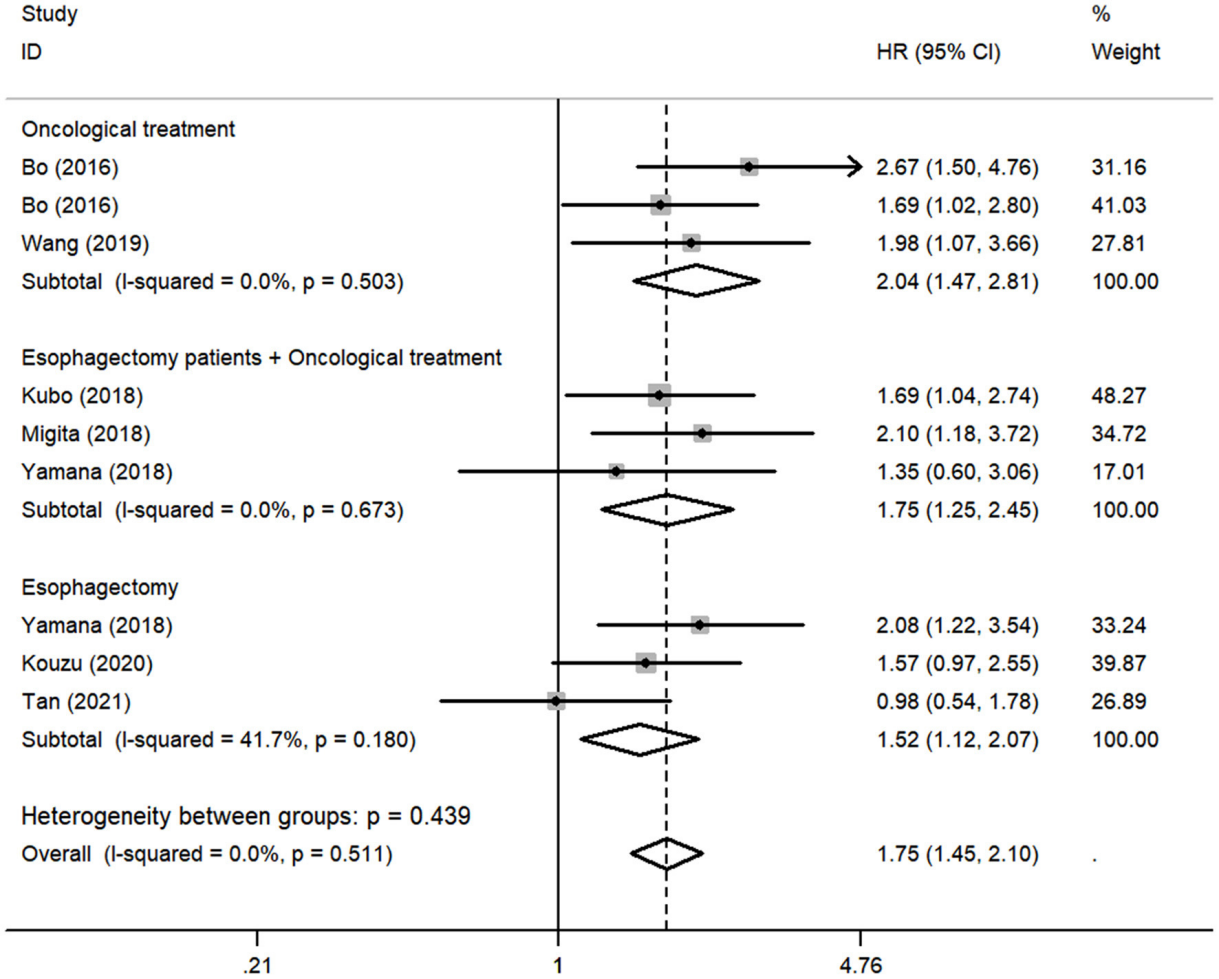
Forest plot showing subgroup analysis of the selected studies about the prognostic significance of GNRI in patients with different treatments.

**Figure 5:**
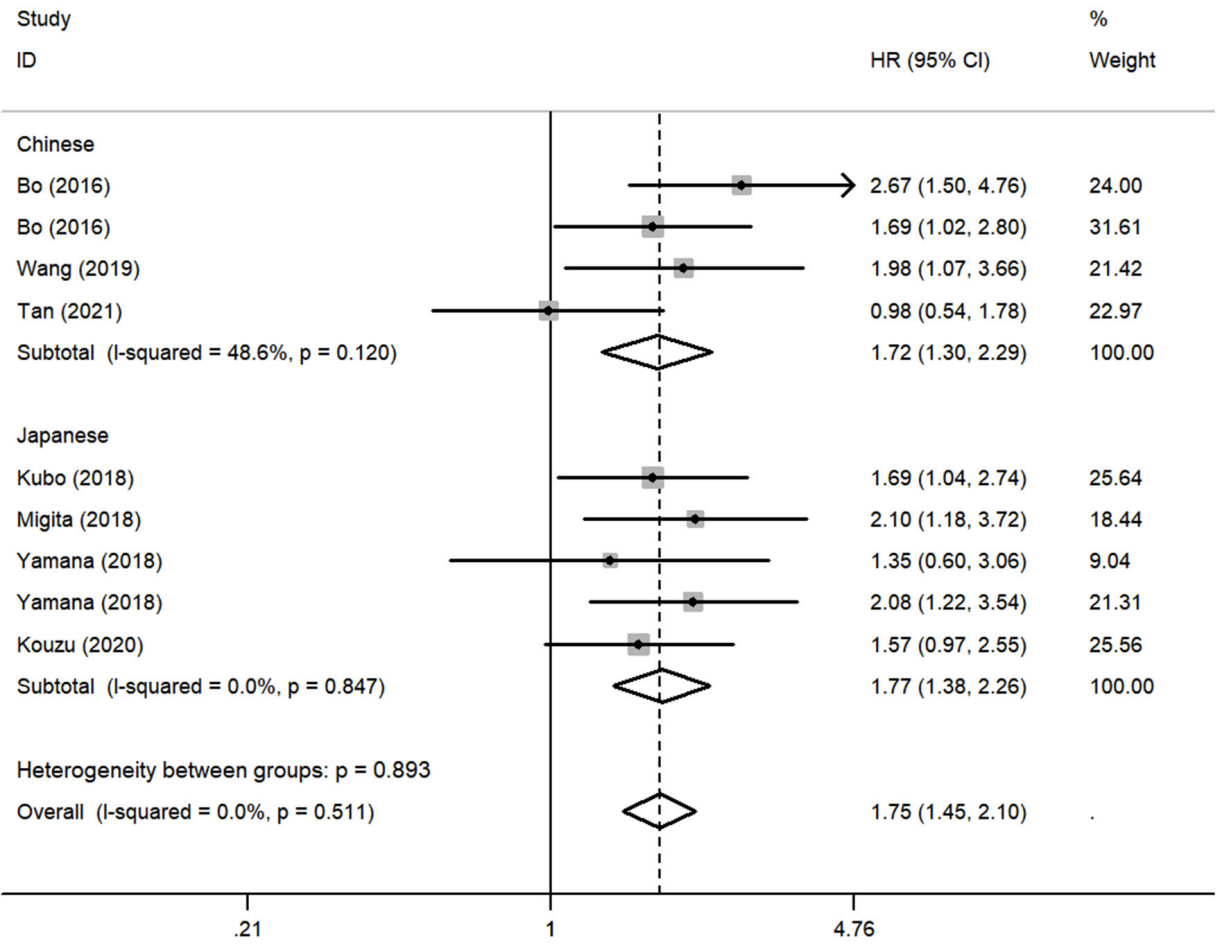
Forest plot showing subgroup analysis of the selected studies about the prognostic significance of GNRI in patients with different populations.

Although there was no obvious heterogeneity among the studies, we still conducted a subgroup analysis to achieve a deeper investigation. The criteria of the subgroups were as follows: cutoff value, therapeutic method, and population. In the subgroup of cutoff value, no heterogeneity was found in studies (*I*^2^ = 0.0%, *P* = 0.714), and a fixed-effects model was applied to the analysis. We concluded that the low GNRI was significantly associated with the worse OS when the cutoff value was set as 92 (HR: 1.86, 95% CI: 1.48–2.34, *P* < 0.01). When setting the cutoff value as 98, the low GNRI was also associated with poorer OS (HR: 1.86, 95% CI: 1.27–2.71, *P* < 0.01), without any heterogeneity (*I*^2^ = 0.0%, *P* = 0.578) (Figure 3). In the subgroup of patient treatment, low GNRI and poor OS were statistically significantly associated with patients who underwent surgical therapy (HR: 1.52, 95% CI: 1.12–2.07, *P* < 0.05; fixed-effects model), oncological treatment (HR: 2.04, 95% CI: 1.47–2.81, *P* < 0.05; fixed-effects model), and esophagectomy with oncological treatment (HR: 1.75, 95% CI: 1.25–2.45, *P* < 0.05; fixed-effects model) (Figure 4). In the subgroup of the population, we found that low GNRI significantly related to poor prognosis in both Chinese patients (HR: 1.72, 95% CI: 1.30–2.29, *P* < 0.01; fixed-effects model) and Japanese patients (HR: 1.77, 95% CI: 1.38–2.26, *P* < 0.01; fixed-effects model), and there was no heterogeneity in the data (Figure 5), so we used the fixed-effects model for analysis. These two subgroup analyses both observed that low GNRI was associated with poor OS regardless of population.

Two of the included studies had investigated the association between GNRI value and postoperative complication rate using odds ratio (OR). The ORs of Kubo et al. (17) and Migita et al. (18) were 1.467 (95% CI: 0.414–5.196, *P* = 0.550) and 1.660 (95% CI: 0.771–3.576, *P* = 0.196), respectively. The pooled OR was 1.606 (95% CI: 0.883–3.094, *P* = 0.157), which indicated that low GNRI does not increase the risk of postoperative complications (Table 3).

**Table 3:** The relationships between the GNRI and postoperative complication rate.

**Study**	**Year**	**GNRI**	
		**OR (95%CI)**	***P***
Kubo et al. (17)	2018	1.467 (0.414–5.196)	0.550
Migita et al. (18)	2018	1.660 (0.771–3.576)	0.196
Pooled OR		1.606 (0.883–3.094)	0.157

*OR, odds ratio; GNRI, geriatric nutritional risk index*.

### Sensitivity Analysis and Publication Bias

**Figure 6:**
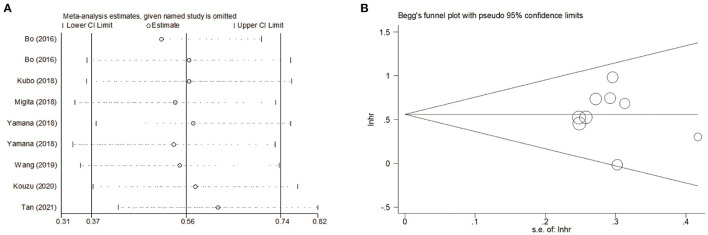
**(A)** Sensitivity analysis for meta-analysis of GNRI. **(B)** Funnel plots of publication bias for meta-analysis of GNRI.

To assess the stability and reliability of the primary analysis, sensitivity analysis was utilized through sequential removal of each study. The result showed that the survival outcome of the prime analysis was not influenced by removing any single study, even when drawing the study with relatively low quality (Figure 6A). Moreover, the hidden publication bias was tested using Begg's test. A symmetrical appearance was checked in the funnel plot (Figure 6B). The *P*-value of Begg's test was 0.759. Therefore, no notable publication bias was found in the meta-analysis.

## Discussion

The nutritional risk index (NRI), combining serum albumin and BMI, was described by Buzby (22) for the first time. Patients with EC often have difficulty in per OS nutrition due to postoperative anastomotic stenosis, which is often accompanied by symptoms of malnutrition. The most common manifestations are weight loss and reduced albumin. However, a single nutritional index cannot fully reflect the nutritional status of patients with EC. Recently, various nutritional indexes [such as GNRI and skeletal muscle mass index (23)] have emerged in evaluating the nutritional status of patients with EC, which have better manifestation than traditional NRI. GNRI is a nutritional indicator combined with both serum albumin, present, and ideal body weight, which could accurately reflect the nutritional level of patients and provide more comprehensive nutritional support treatment, thereby improving the accuracy of predicting the prognosis of patients with EC (24). The index has been wildly applied to evaluate the malnutrition status and severity degree of postoperative complications of hospitalized adults. However, patients with EC, especially elderly patients, were usually suffering from malnutrition and weight loss due to insufficient nutritional intake (25). As a result, the concept of GNRI was introduced by Bouillanne et al. for the first time in 2005. GNRI was an omnibus index, which took ideal body weight into consideration at the basis of NRI. Therefore, GNRI was advantaged in evaluating the nutritional status of senile patients. The GNRI score was reported as an independent indicator of morbidity and mortality in patients with chronic heart failure (26) and sepsis (27) in previous research. In recent years, GNRI was applied to predict the long-term outcomes of upper digestive cancer, such as EC and gastric cancer (28). For EC, the amount of study related to GNRI and OS was limited, and the predictive efficiency of GNRI was not clear. Thus, this meta-analysis was conducted to explore the influence of GNRI on the survival outcomes of EC. To the best of our knowledge, no meta-analysis of this topic has ever been performed before us.

We totally included 11 studies in this meta-analysis, containing 1785 patients. The cutoff value of GNRI in the studies was divided into two categories as follows: GNRI <92 and GNRI > 98. Only two included studies of Hirahara et al. and Tan et al. set the cutoff value as GNRI = 97.1 and GNRI = 96.6, lacking studies setting GNRI in the same standard, and we did not include these two studies into subgroup analysis. According to the results of the cutoff value subgroup analysis, the pooled HR showed that a lower level of GNRI had a significant adverse influence on the OS of patients with EC. Meanwhile, we could easily get the same conclusion from the other two subgroups' results according to the pooled HRs. Bo et al. first conducted the study to explore the relationship between GNRI and 5-year OS of EC, indicating that higher HR was related to lower GNRI (1.69 for 92–98 vs. >98; 2.67 for <92 vs. >98) (14). Thereafter, several similar studies were carried out. In the study by Migita et al. (18), the HR was 2.10 with 95% CI 1.18–3.72 (<98 vs. >98). For the studies by Kubo et al. (17) and Yamana et al. (21), the HRs were 1.687 (95% CI: 1.038–2.742) and 1.35 (95% CI: 0.59–3.03). In addition, Hirahara et al. (15) used CSS as the survival outcome to evaluate the impact of GNRI on patients with EC in different EC stages. However, the sample sizes of the previous studies were not large enough. Thus, we pooled these studies into this meta-analysis. The result suggested that GNRI was potential to be a prognostic factor of long-term OS of patients with EC.

In past studies, low GNRI has been reported to be associated with the prognosis of colorectal cancer (29), non-small cell lung cancer (30), lymphoma, nasopharyngeal cancer (30), lymphoma, and nasopharyngeal cancer (31, 32). Consistently, our study confirms that GNRI is closely associated with the long-term prognosis of EC. Cancer-associated malnutrition plays an essential and multifaceted role in tumor progression. The exact mechanism between malnutrition and tumor in patients with EC was still undefined. However, it has increasingly been acknowledged that cancer-caused nutritional disorders, such as cachexia and sarcopenia (33, 34), are admitted to be irreversible outcomes of the interaction between host and tumor (35). Moreover, nutritional disorders caused by a tumor also raise the risk of infectious complications in surgery, weaken the efficacy of chemoradiotherapy, and increase the incidence of side effects of adjuvant therapy (36), which are closely related to the patient's prognosis.

This analysis had several limitations. First, no well-designed RCTs but only retrospective cohort studies were brought into the study, probably causing reduced statistical effectiveness. In addition, the total amount of patients in studies was only 1785, the remaining suffering from a limited sample size. Second, most included studies only focus on the OS. This may not comprehensively and systematically reflect the GNRI impact on EC prognosis. Other long-term results, such as recurrence-free survival (RFS), progression-free survival (PFS), and disease-free survival (DFS), should be taken into account. Third, the therapy strategies were not all the same in the included cohort studies, although no apparent heterogeneity was found. Fourth, most researchers have used different cutoff values in their studies to define the GNRI level, lacking uniform criteria for the cutoff value of GNRI in different studies. The pooled survival outcomes may deviate from the actual value. Finally, the patients' population was all from the Asian group; no western research was included, which may lead to a selection bias in the patients' races to some degree. Considering all the limitations listed above, which might affect the validity of the results, the conclusion is not persuasive enough and needs to be refined. Thus, more well-conducted studies with large sample sizes, especially RCTs, were urgently needed to confirm and update our conclusion. Meanwhile, the following studies should complete different survival outcomes, and patients from different races should also be included so that the subgroup analysis could better elucidate the correlation between GNRI and EC prognosis.

## Conclusion

Overall, a lower level of GNRI was associated with poor survival outcomes. GNRI was a potential independent prognostic indicator for patients with EC. Meanwhile, more high-quality studies are needed to confirm the findings.

## Data Availability Statement

The original contributions presented in the study are included in the article/supplementary material, further inquiries can be directed to the corresponding author/s.

## Author Contributions

YYu conceptualized the study, revised the manuscript, and supervised the study. JZ, PF, and XL conceptualized the study, drafted the manuscript, and made the figures. SL, XX, YG, QS, HZ, YYa, and XZ collected the literature and revised the manuscript. All authors contributed to this study and approved the submitted version.

## Funding

This work was supported by the National Nature Science Foundation of China (81970481 and 82000514) key projects of Sichuan Provincial Department of Science and Technology (22ZDY1959 and 2021YFS0222), 1.3.5 Project for Batch of Excellence, West China Hospital, Sichuan University (2020HXFH047, ZYJC18010, 20HXJS005, and 2018HXFH020), and China Postdoctoral Science Foundation (2020M673241).

## Conflict of Interest

The authors declare that the research was conducted in the absence of any commercial or financial relationships that could be construed as a potential conflict of interest.

## Publisher's Note

All claims expressed in this article are solely those of the authors and do not necessarily represent those of their affiliated organizations, or those of the publisher, the editors and the reviewers. Any product that may be evaluated in this article, or claim that may be made by its manufacturer, is not guaranteed or endorsed by the publisher.
